# Special Issue: Gene Conversion in Duplicated Genes

**DOI:** 10.3390/genes2020394

**Published:** 2011-06-17

**Authors:** Hideki Innan

**Affiliations:** Graduate University for Advanced Studies, Hayama, Kanagawa 240-0193, Japan; E-Mail: innan_hideki@soken.ac.jp

Gene conversion is an outcome of recombination, causing non-reciprocal transfer of a DNA fragment. Several decades later than the discovery of crossing over, gene conversion was first recognized in fungi when non-Mendelian allelic distortion was observed. Gene conversion occurs when a double-strand break is repaired by using homologous sequences in the genome. In meiosis, there is a strong preference to use the orthologous region (allelic gene conversion), which causes non-Mendelian allelic distortion, but paralogous or duplicated regions can also be used for the repair (inter-locus gene conversion, also referred to as non-allelic and ectopic gene conversion). The focus of this special issue is the latter, interlocus gene conversion; the rate is lower than allelic gene conversion but it has more impact on phenotype because more drastic changes in DNA sequence are involved.

This special issue consists of 10 review papers covering various aspects of interlocus gene conversion. Hastings [1] provides a review on the basic mechanisms of gene conversion in meiotic cells. Gene conversion occurs through the repair process of a double-strand break with the formation of a D-loop. Gene conversion also occurs in mitosis; the mechanisms should be the same but different proteins may be involved. Kurosawa and Ohta [2] review gene conversion in somatic cells with special emphasis on the role to generate functional variation in a gene family, which is recognized as an important mechanism for immunoglobulin (Ig) genes. In the chicken genome, there are a number of pseudogenes around a functional Ig gene, and small pieces of fragments in these pseudogenes are frequently transferred to the functional gene in mitosis, thereby creating a novel function. Another article by Lin, Mikawa and Shibata [3] uniquely focuses on gene conversion between mitochondrial DNA. There are a huge number of copies of mtDNA in a cell, where the rate of mutation is exceptionally high. Heteroplasmy of mtDNA caused by such a high mutation rate is potentially deleterious, and gene conversion works to homogenize it. Thus, gene conversion occurs from meiosis to mitosis, even in mitochondria, and the molecular mechanism should be similar; basically, a double-strand break is repaired by a homologous sequence. There is a strong preference to use a sequence of high identity. This makes the rate of gene conversion between allelic regions higher than that between duplicates with a certain level of divergence. There also are many factors (e.g., physical location, MEPS: minimal efficient processing segment) to determine the rate of gene conversion, and extensive studies have been carried out to quantitatively evaluate the effect of such factors, which is reviewed by Mansai, Kado and Innan [4].

As well as other mutational mechanisms, gene conversion causes changes in genomic DNA, which potentially have an effect on phenotype. Such phenotypic changes are the driving force of evolution, and Ohta [5], who made the fundamental theoretical framework of the molecular evolution of gene families, provides an interesting historical review on evolutionary research. Gene conversion between duplicated copies in a gene family basically homogenizes genetic variation within the family, so that copy members in the family can maintain high levels of sequence homology long-term (referred to as concerted evolution). Concerted evolution was first recognized in the ribosomal gene family in *Xenopus*, and a number of individual gene-based demonstrations of concerted evolution followed, but its impact on a genomic scale was not clear in the 20th century. Since the genome sequences of various species became available, it has been demonstrated that concerted evolution by gene conversion plays significant roles in many eukaryotes, especially in early stages of the evolution of gene duplicates. Arguello and Connallon [6] provide a wide overview of research on gene conversion in *Drosophila*, which has a long history of research on repetitive DNA including ribosomal gene families since the pre-molecular biology era. Nematodes also provide an intriguing resource for studying gene conversion because they are known as duplication-rich species, as reviewed by Katju and Bergthorsson [7]. Plants are unique in that many species experienced multiple rounds of genome duplication. Wang and Patterson [8] provide recent views on concerted evolution between duplicates from polyploidy events in angiosperms revealed by their genomic sequences.

Gene conversion in humans is of particular interest due to medical reasons. There are a number of pseudogenized duplicates in the human genome, which usually accumulate deleterious mutations. Gene conversion causes genetic diseases when such deleterious mutations are transferred to the functional copy, as reviewed by Chen, Férec and Cooper [9]. Such deleterious gene conversion is likely eliminated from the population, but there would be situations where selection works for gene conversion. Fawcett and Innan [10] review theoretical works to understand the joint action of gene conversion and selection in the population genetic process and introduce several examples that exhibit clear evidence for natural selection on gene conversion.

I am happy to see that this special issue includes a wide range of topics, from the mechanisms to molecular evolution. It also covers historical reviews and very recent updates from genomic sequencing. I hope this special issue enhances further research on gene conversion. I would like to thank all authors and reviewers who contributed to this special issue.

## References

[1] Hastings P.J. (2010). Mechanisms of ectopic gene conversion. Genes.

[2] Kurosawa K., Ohta K. (2011). Genetic diversification by somatic gene conversion. Genes.

[3] Ling F., Mikawa T., Shibata T. (2011). Enlightenment of yeast mitochondrial homoplasmy: Diversified roles of gene conversion. Genes.

[4] Mansai S.P., Kado T., Innan H. (2011). The rate and tract length of gene conversion between duplicated genes. Genes.

[5] Ohta T. (2010). Gene conversion and evolution of gene families: An overview. Genes.

[6] Arguello J.R., Connallon T. (2011). Gene duplication and ectopic gene conversion in *Drosophila*. Genes.

[7] Katju V., Bergthorsson U. (2010). Genomic and population-level effects of gene conversion in Caenorhabditis paralogs. Genes.

[8] Wang X.-Y., Paterson A.H. (2011). Gene conversion in Angiosperm genomes with an emphasis on genes duplicated by polyploidization. Genes.

[9] Chen J.-M., Férec C., Cooper D.N. (2010). Gene conversion in human genetic disease. Genes.

[10] Fawcett F.A., Innan H. (2011). Neutral and non-neutral evolution of duplicated genes with gene conversion. Genes.

